# A novel signature constructed by mitochondrial function and cell death-related gene for the prediction of prognosis in bladder cancer

**DOI:** 10.1038/s41598-024-65594-0

**Published:** 2024-06-25

**Authors:** Zhiwei Yan, Yunxun Liu, Minghui Wang, Lei Wang, Zhiyuan Chen, Xiuheng Liu

**Affiliations:** 1 Department of Urology, Renmin Hospital of Wuhan University, Wuhan, 430060 China; 2 Institute of Urologic Disease, Renmin Hospital of Wuhan University, Wuhan, 430060 China

**Keywords:** Mitochondrial function, Cell death, BLCA, Prognosis, ICI, Single-cell, Cancer, Computational biology and bioinformatics, Urology

## Abstract

Bladder urothelial carcinoma (BLCA) presents a persistent challenge in clinical management. Despite recent advancements demonstrating the BLCA efficacy of immune checkpoint inhibitors (ICI) in BLCA patients, there remains a critical need to identify and expand the subset of individuals who benefit from this treatment. Mitochondria, as pivotal regulators of various cell death pathways in eukaryotic cells, exert significant influence over tumor cell fate and survival. In this study, our objective was to investigate biomarkers centered around mitochondrial function and cell death mechanisms to facilitate prognostic prediction and guide therapeutic decision-making in BLCA. Utilizing ssGSEA and LASSO regression, we developed a prognostic signature termed mitochondrial function and cell death (mtPCD). Subsequently, we evaluated the associations between mtPCD score and diverse clinical outcomes, including prognosis, functional pathway enrichment, immune cell infiltration, immunotherapy response analysis and drug sensitivity, within high- and low-risk subgroups. Additionally, we employed single-cell level functional assays, RT-qPCR, and immunohistochemistry to validate the differential expression of genes comprising the mtPCD signature. The mtPCD signature comprises a panel of 10 highly influential genes, strongly correlated with survival outcomes in BLCA patients and exhibiting robust predictive capabilities. Importantly, individuals classified as high-risk according to mtPCD score displayed a subdued overall immune response, characterized by diminished immunotherapeutic efficacy. In summary, our findings highlight the development of a novel prognostic signature, which not only holds promise as a biomarker for BLCA prognosis but also offers insights into the immune landscape of BLCA. This paradigm may pave the way for personalized treatment strategies in BLCA management.

## Introduction

BLCA is responsible for more than 573,000 new cases and approximately 213,000 deaths in 2020, ranking it as the tenth most common cancer and represents a significant cause of morbidity and mortality^1^. Programmed cell death (PCD) is a vital biological process that plays an important role in maintaining tissue homeostasis, development, and immunity. This tightly regulated mechanism allows multicellular organisms to eliminate unwanted or damaged cells in a controlled manner, ensuring proper growth and function. PCD can be divided into apoptotic cell death and non-apoptotic cell death based on morphological characteristics and molecular mechanisms. The former maintains cell membrane integrity and occurs in a caspase dependent manner, while the latter experiences cell membrane rupture and is caspase independent^2^. Classic examples of apoptotic cell death include apoptosis and anoikis, while emerging research has unveiled various non-apoptotic cell death modalities, enriching our understanding of cellular demise mechanisms^3^.

The mitochondria, as the central hub of cellular metabolism, undergo structural and functional changes closely associated with cellular fate^4^. Mitochondrial outer membrane permeabilization (MOMP) is a critical event in apoptosis initiation^5,6^. Even in instances of mitochondrial dysfunction, inhibition of caspase activity in the presence of MOMP can still lead to non-apoptotic cell death, highlighting the paramount importance of OMM integrity as the primary determinant of cellular fate^7^. Mitochondria also play a crucial role in some newly discovered forms of non-apoptotic PCD. Necroptosis is a form of PCD with the same morphological features as necrosis. It has been shown that progressive mitochondrial dysfunction may predispose cells to necroptosis^8^. Ferroptosis, characterized by iron-dependent lipid peroxidation, represents a novel mode of PCD. Given the pivotal role of mitochondria in iron utilization, catalysis, anabolic pathways, and iron homeostasis regulation, they stand at the forefront of ferroptosis regulation^9^. Similarly, cuproptosis, a copper-dependent form of cell death, is driven by mitochondrial stress and injury^10^. In essence, mitochondrial function intricately intertwines with the mechanisms of PCD, forming an inseparable entity.

It is widely acknowledged that tumor cells possess the capability to evade PCD, primarily through the suppression of the immune clearance mechanism. Consequently, reversing the immunosuppressive state within the body emerges as pivotal in facilitating PCD of tumor cells, thus constituting a crucial direction in tumor immunotherapy research^11^. In recent years, the advent of ICI grounded on this premise has heralded a significant breakthrough in BLCA treatment, eliciting the anticancer potential of T cells by obstructing the interaction between programmed cell death protein 1 (PD-1) and cytotoxic T-lymphocyte antigen 4 (CTLA-4) with their cognate ligands^12^. Presently, pembrolizumab and atezolizumab have garnered approval from the US Food and Drug Administration (FDA) for first-line therapy in advanced BLCA patients ineligible for platinum-based chemotherapy^13^. Nonetheless, delineating the target beneficiary cohort remains a formidable challenge^14^. Intriguingly, PCD assumes a pivotal role in shaping ICI immunotherapy efficacy. Studies have observed that CD8 T cells can inhibit tumor cells by inducing ferroptosis and pyroptosis, while concurrently modulating T cell functionality through ferroptosis, thus exerting a pivotal impact on tumor immunotherapy^15^. Consequently, investigations probing this intricate interplay promise to deepen insights into the underlying oncogenic mechanisms of BLCA and offer novel avenues for refining BLCA immunotherapeutic strategies.

In this study, we defined a new concept, mitochondrial function and cell death (mtPCD), and developed a prognostic signature associated with mtPCD to predict the prognosis of BLCA patients. Notably, we verified that the mtPCD signature could improve the accuracy of BLCA prognosis prediction. In addition, we performed functional analysis, tumor immune microenvironment (TIME) and immunotherapy response analysis, drug sensitivity analysis, and single-cell analysis to more fully understand the significance of the mtPCD score. Ultimately, our findings may point to new directions for the diagnosis and treatment of BLCA.

## Results

### Establishment of the mtPCD-related prognostic signature

**Figure 1:**
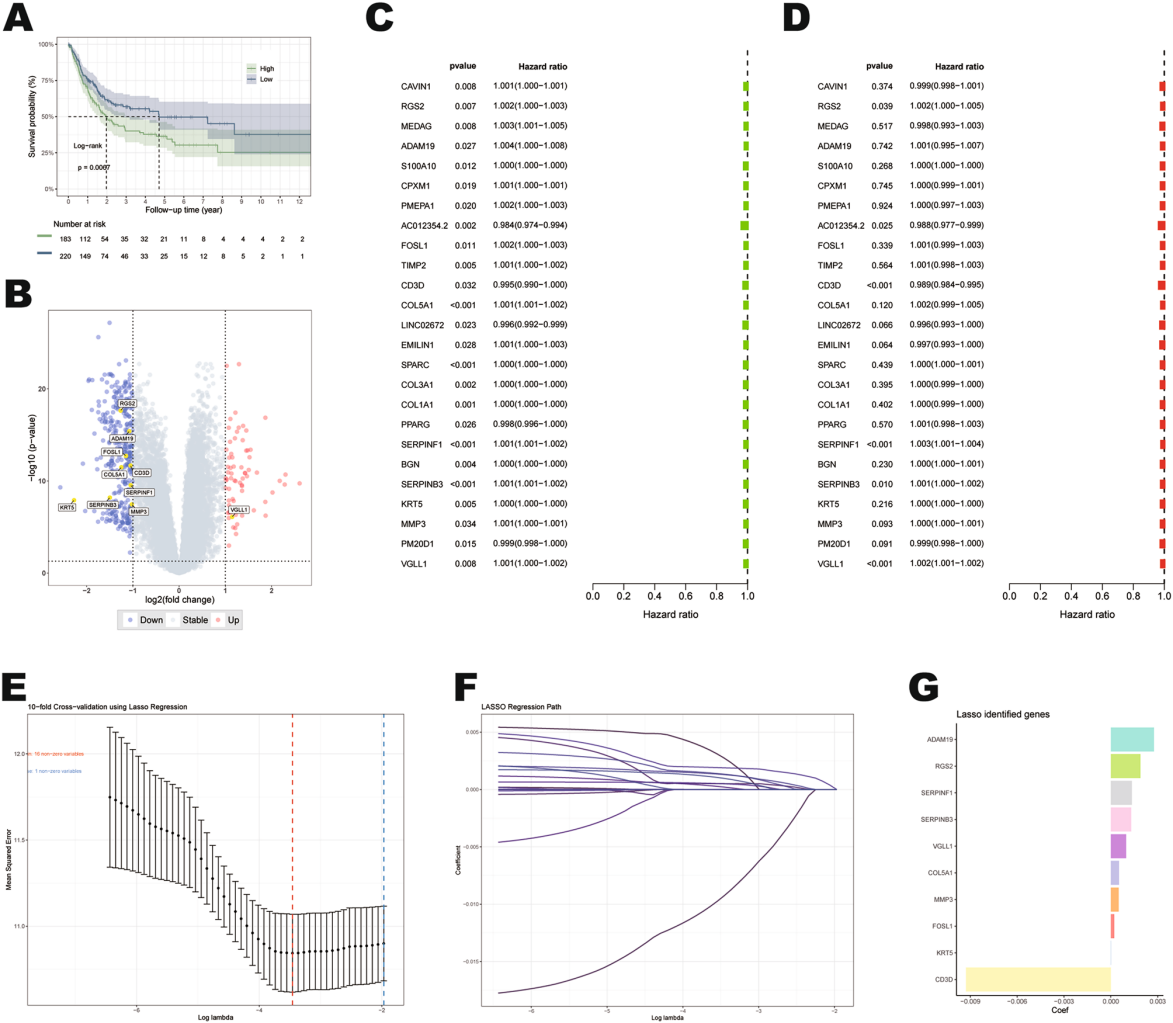
Establishment of the prognostic signature related to mitochondrial programmed cell death (mtPCD). (**A**) Survival analysis based on ssGSEA scores. (**B**) Differential expression of mtPCD-related genes between high score group and low score group. (**C**, **D**) Forest plot of the top 25 genes in univariate Cox analysis. (**E**–**G**) Identification of 10 mtPCD-related genes and their corresponding coefficient values through LASSO regression.

To explore the relationship between mtPCD and BLCA, we performed ssGSEA to assess the enrichment scores of the mtPCD gene set in TCGA samples and divided the cohort into mtPCD high and low subgroups (Supplementary Table S1). According to the Kaplan–Meier curve, patients in the high subgroup had a significantly reduced survival time (*P* < 0.01; Fig. 1A). Subsequently, 402 differentially expressed genes were identified between the high and low mtPCD subgroups. (Fig. 1B), and then finally obtained 25 prognostically relevant candidate genes by univariate Cox analysis (Fig. 1C,D). In order to identify the core mtPCDRGs, we used LASSO regression analysis (Fig. 1E,F), and finally selected 10 core genes as mtPCD characteristic biomarkers among the 25 mtPCDRGs (Fig. 1G). The risk score was calculated as follows: risk score = (0.001896 × Exp RGS2) + (0.002757 × Exp ADAM19) + (0.000217 × Exp FOSL1) + (− 0.00928 × Exp CD3D) + (0.000514 × Exp COL5A1) + (0.001341 × Exp SERPINF1) + (0.001304 × Exp SERPINB3) + (4.74E−06 × Exp KRT5) + (0.0005 × Exp MMP3) + (0.000975 × Exp VGLL1).

### Validation of the accuracy of prognostic signature

**Figure 2:**
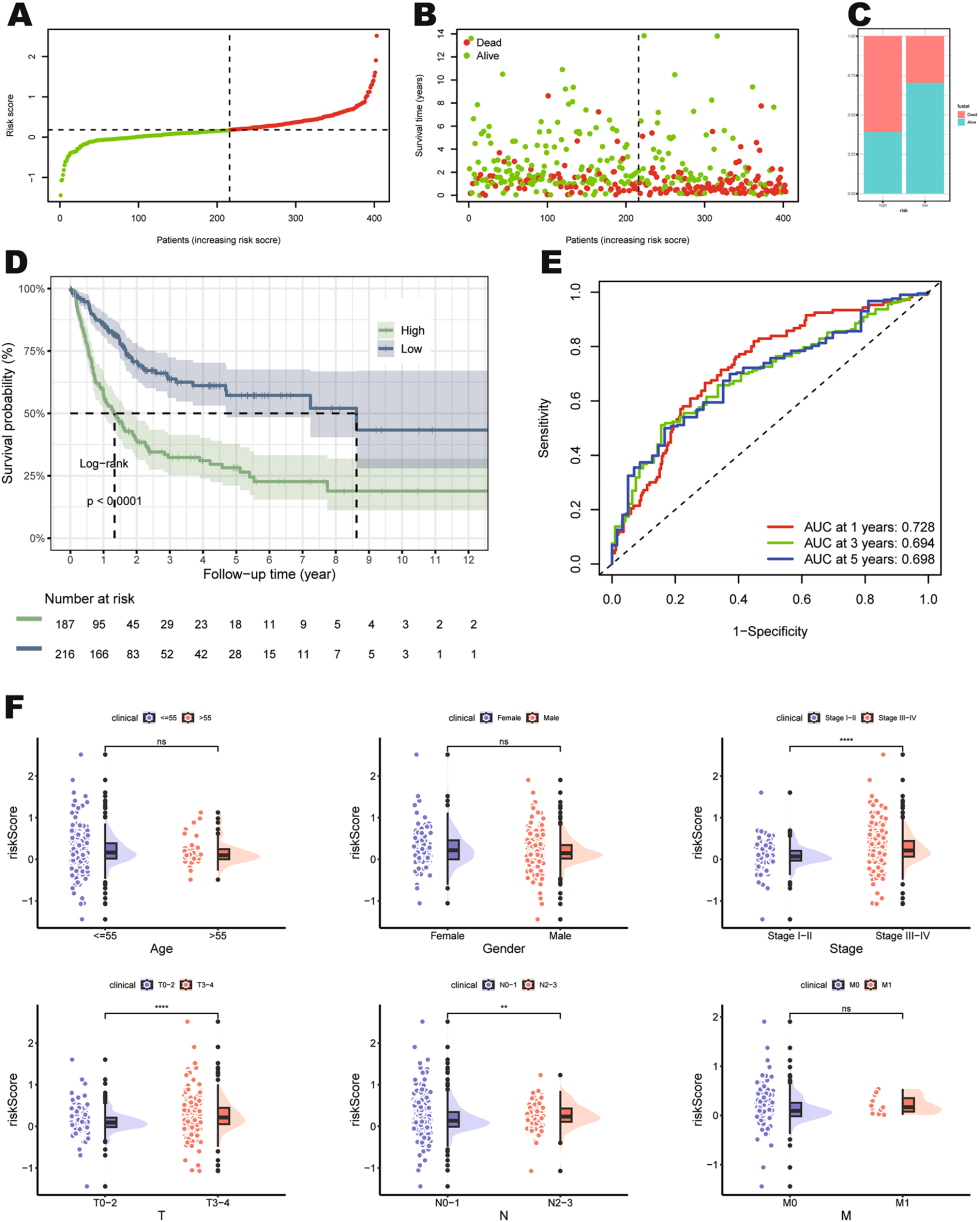
Validation and clinical relevance analysis of the prognostic signature. (**A**–**C**) Distribution of patients and the proportion of individuals in different survival states in the TCGA-BLCA cohort. (**D**) Kaplan Meier curves of the TCGA-BLCA cohort. (**E**) ROC curves at 1-, 3-, and 5- year in the TCGA-BLCA cohort, with respective AUC of 0.728, 0.694, and 0.698. (**F**) Demonstrates the risk score distribution based on different clinical pathological features.

We next assessed and validated the prognostic signature. Firstly, the TCGA-BLCA cohort was stratified into high- and low-risk groups based on the median of the mtPCD riskScore. The high-risk group exhibited a significantly higher mortality rate compared to the low-risk group (TCGA-BLCA cohort: Fig. 2A–C; GSE13507 cohort: Supplementary Fig. S1A–C; GSE32894 cohort: Supplementary Fig. S2A–C)***.*** The survival curve analysis revealed that patients in the mtPCD high- risk group exhibited poorer survival outcomes (TCGA-BLCA cohort: Fig. 2D; GSE13507 cohort: Supplementary Fig. S1D; GSE32894 cohort: Supplementary Fig. S2D). To assess the stability and reliability of the prognostic signature, we generated Receiver Operating Characteristic (ROC) curves for OS at 1, 3, and 5 years. In the TCGA-BLCA cohort (Fig. 2E), the area under the curve (AUC) values were 0.728, 0.694, and 0.698, respectively. Upon further validation using two additional validation cohorts, the utility of the signature was consistent with the training set results. In the GSE13507 cohort (Supplementary Fig. S1E), the AUC values were 0.678, 0.644, and 0.620, respectively. In the GSE32894 cohort (Supplementary Fig. S2E), the AUC values were 0.705, 0.760, and 0.725, respectively. Thus, the model exhibits good predictive performance. Furthermore, there were significant differences between tumor stage (stage I-II and stage III-IV), T-stage (T0-T2 and T3-T4), N-stage (N0-N1 and N2-N3) and risk score in the TCGA cohort (Fig. 2F). In the GSE13507 cohort, significant differences were found among stage (stage I-II and stage III-IV), T stage (T0-T1 and T2-T4), and risk score (Supplementary Fig. S1F). In the GSE32894 cohort, significant differences were observed among T stage (T0-T1 and T2-T4), grade (G1 and G2-G3) and risk score (Supplementary Fig. S2F). These findings indicate that the high-risk group is closely associated with the malignancy of tumors.

### Comparison of gene expression-based prognostic signatures in BLCA

**Figure 3:**
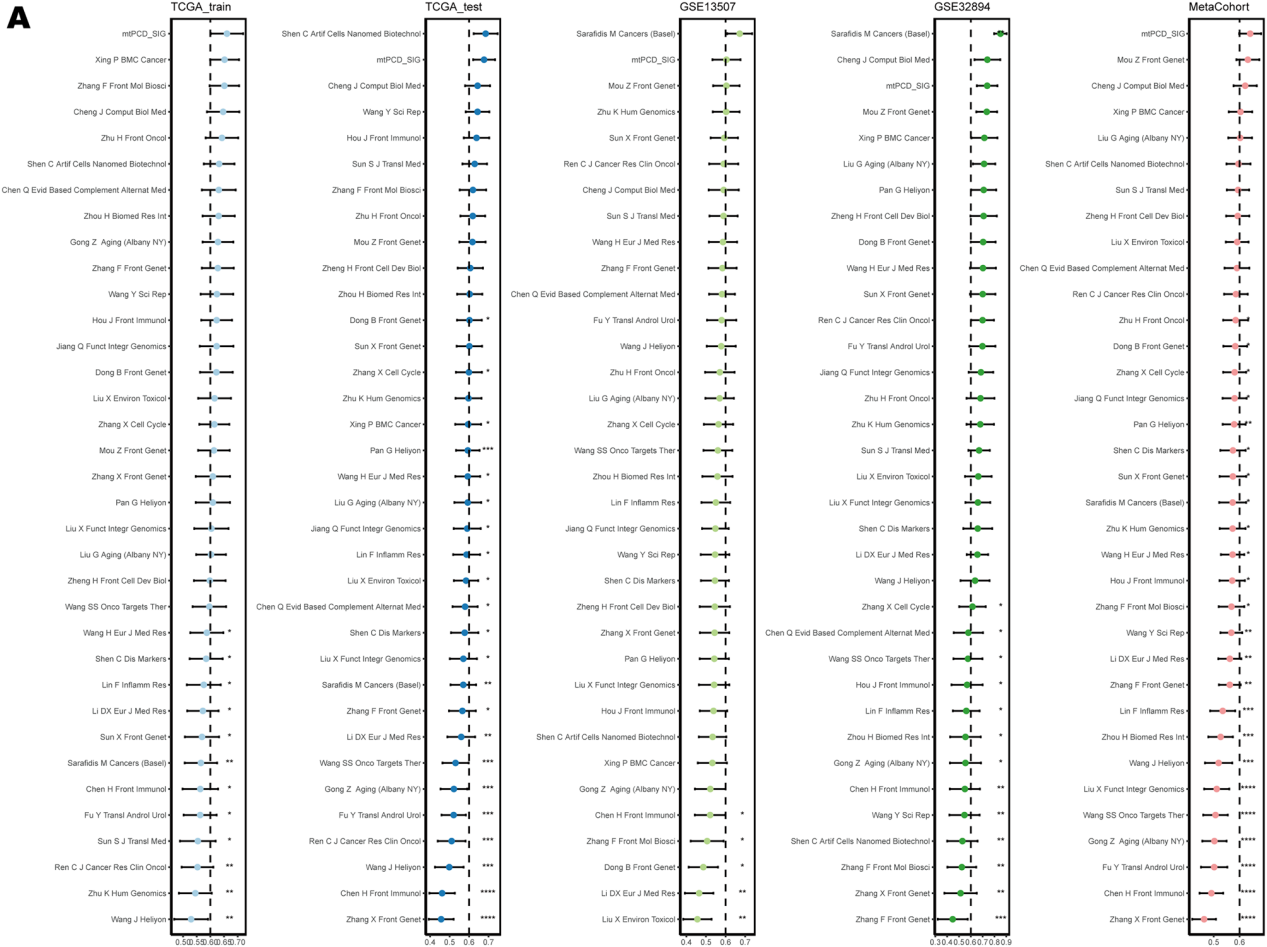
Comparison of gene expression-based prognostic signatures in BLCA. (**A**) Analysis of mtPCD and 35 published models by C-index in TCGA-BLCA, GSE13507, GSE32984, and meta-cohort cohorts.

With the development of the bioinformatics era, a large number of BLCA-based predictive and prognostic signatures have been developed. In order to distinguish the advantages of mtPCD from other BLCA-related prognostic signatures, we have collected the published BLCA-related mRNA signatures in PubMed, and these collected signatures are related to various biological processes, such as tumor microenvironment, immune response, lipid metabolism, ferroptosis, hypoxia, epithelial-mesenchymal transition and drug sensitivity. Due to the severe lack of miRNA import in the validation dataset of some of the microarrays, prognostic signatures of miRNAs were excluded. Eventually, 35 signatures were collected (Supplementary Table S2). Next, we compared the C-index between mtPCD and other signatures, and for models lacking coefficients, reconstruction was performed using the Cox model. We observed that most models perform well on their own training datasets but show poor performance on other datasets. In contrast, mtPCD consistently ranks in the top three positions across all datasets, demonstrating robust performance (Fig. 3A).

### Construction and evaluation of the nomogram

**Figure 4:**
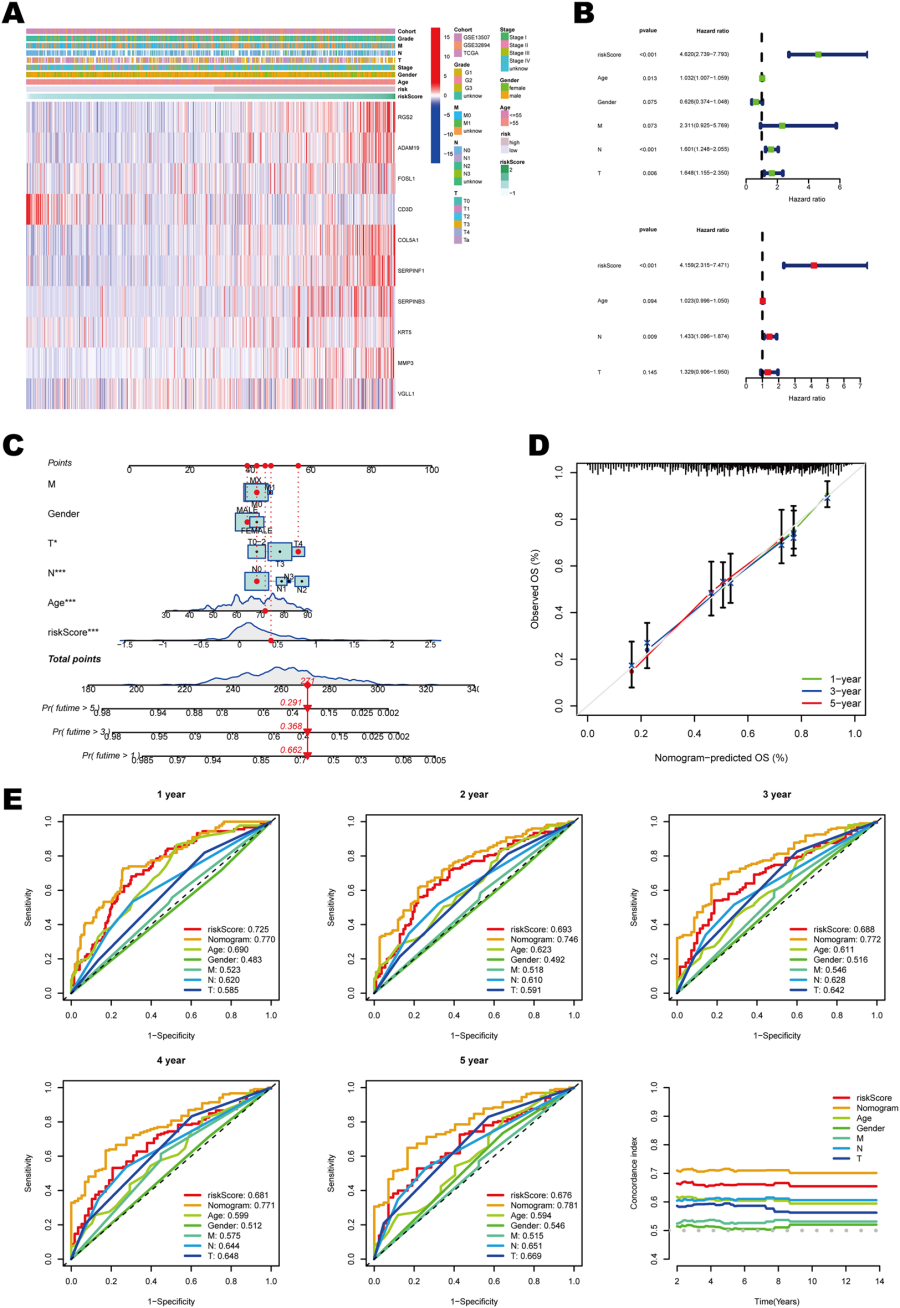
Construction and performance validation of the nomogram. (**A**) Distribution of signature genes and clinical-pathological characteristics between high- and low-risk groups in three datasets. (**B**) Univariate and multivariate Cox regression analysis to screen potential prognostic factors for overall survival. (**C**) The nomogram for predicting the probability of overall survival at 1-, 3-, and 5-year. (**D**) Calibration curves for the nomogram. (**E**) Verification of diagnostic accuracy by constructing ROC curves and time-dependent C-index curves for various indicators in different time periods.

The expression profiles of 10 mtPCD signature genes in high- and low-risk groups, as well as their distribution across various clinical pathological features, were visualized in the form of a heatmap (Fig. 4A). To validate the clinical application of the mtPCD signature, we integrated the clinical information of the patients, including survival time, survival status, age, gender, and TNM staging (Supplementary Table S3). Univariate and multivariate Cox regression analyses were used to assess the effects of clinical characteristics and mtPCD on BLCA survival. The results showed that risk score and N stage could be used as independent prognostic factors (Fig. 4B). Using these predictors, we designed a nomogram (Fig. 4C) and published the predictive model online (https://urology-yanzw.shinyapps.io/DynNomapp/) to estimate the 1-, 3-, and 5-year survival probabilities of BLCA patients. The calibration curves also demonstrated the predictive accuracy of the nomogram (Fig. 4D). In addition, the AUC values of the nomogram, risk score, and common clinical case characteristics at different time points were displayed in the figure below, the mtPCD and nomogram had more predictive performance than the individual clinical indicators (Fig. 4E). It was observed that the time C-indexes of the nomogram and mtPCD score were the highest among all the variables, suggesting that they were highly predictive of survival outcomes (Fig. 4E). In conclusion, all of these suggest that the nomogram based on mtPCD score have important clinical predictive value for BLCA.

### Analysis of TMIE and immunotherapy response between risk groups

**Figure 5:**
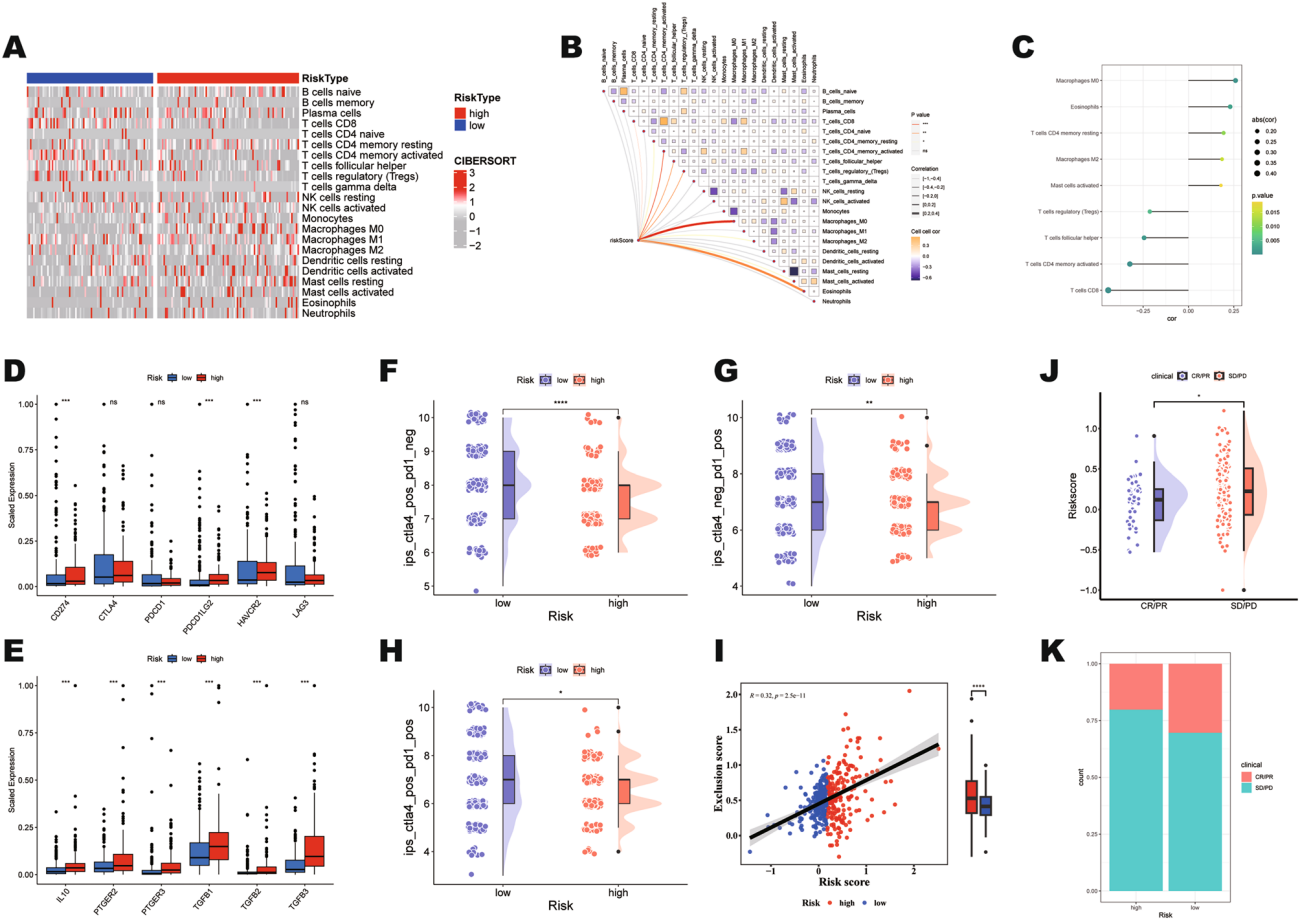
Analysis of immune infiltration and immunotherapy response between risk groups. (**A**) Utilizing the CIBERSORT algorithm to construct a differential immune cell heatmap between two risk subgroups. (**B**) A Heatmap illustrates the correlation between mtPCD risk score and immune infiltrating cells. (**C**) Correlation coefficients of immune infiltrating cells. (**D**) Differential expression of immune checkpoint genes between risk groups. (**E**) Expression of the immune suppressive cytokines between risk groups. (**F**–**H**) The relationship between IPS and risk groups. (**I**) Analysis of the correlation between mtPCD and exclusion scores. (**J**, **K**) The association between risk groups and response to anti-PD-L1 agent treatment.

It is crucial to understand the TMIE landscape in BLCA based on the correlation of mtPCD expression patterns. We used the CIBERSORT algorithm to calculate the differences in immune cell infiltration between the two mtPCD risk groups (Fig. 5A). To further understand the impact of mtPCD on the TIME, correlations between mtPCD score and various immune cell populations within TIME were analyzed. The results showed that a total of 9 immune cells exhibited expression differences between the two risk subgroups. Specifically, in the high-risk group, infiltration levels of CD4 resting memory T cells, M0 macrophages, M2 macrophages, activated mast cells, and eosinophils were significantly increased. Conversely, the low-risk group exhibited accumulation of numerous T cells, including CD8 T cells, CD4 activated memory T cells, follicular helper T cells, and regulatory T cells (Fig. 5B). The mtPCD score positively correlated markedly with tumor immune cells, while showing a remarkable negative correlation with T cells (Fig. 5C). In particular, the higher the mtPCD score, the higher the expression of M0 macrophages and M2 macrophages, and the lower the expression of T cell infiltration. Thus, the high-risk group may be associated with an immunosuppressive phenotype that promotes tumor survival. Common immune checkpoints were compared between two groups. The results showed that CD274 (PD-L1), PDCD1LG2 (PD-L2) and HAVCR2 (TIM-3) were significantly upregulated in high-risk group compared to the counterpart (Fig. 5D). Additionally, we observed notable upregulation of chemokines involved in the immune suppression process (IL10, PTGER2, PTGER3, TGFB1, TGFB2, and TGFB3) in the high-risk group (Fig. 5E). These data suggest that high-risk patients exhibit inertia in antitumor immunities, which might contribute to their poor prognosis. The efficacy of mtPCD signature in predicting immunotherapy response for BLCA patients was evaluated using the Immunophenoscore (IPS), a measure of how well immunotherapy works for patients in the TCGA group. The IPS for PD-1 and CTLA-4 inhibitors in the low-risk group was significantly higher than in the high-risk group, suggesting that patients with low-risk may probably respond better to immunotherapy (Fig. 5F–H). Based on TIDE scores, we found a positive correlation between high-risk groups and exclusion scores (Fig. 5I). Furthermore, we confirmed the effectiveness in the IMvigor210 dataset, which showed that the low-risk group responded better to anti-PD-L1 agent (atezolizumab) (Fig. 5J,K). These results not only underscore the favorable response to immune therapy in the low-risk group but also highlight the prognostic value of these features in predicting immune therapy outcomes in BLCA patients.

### The underlying biological function of mtPCD groups

**Figure 6:**
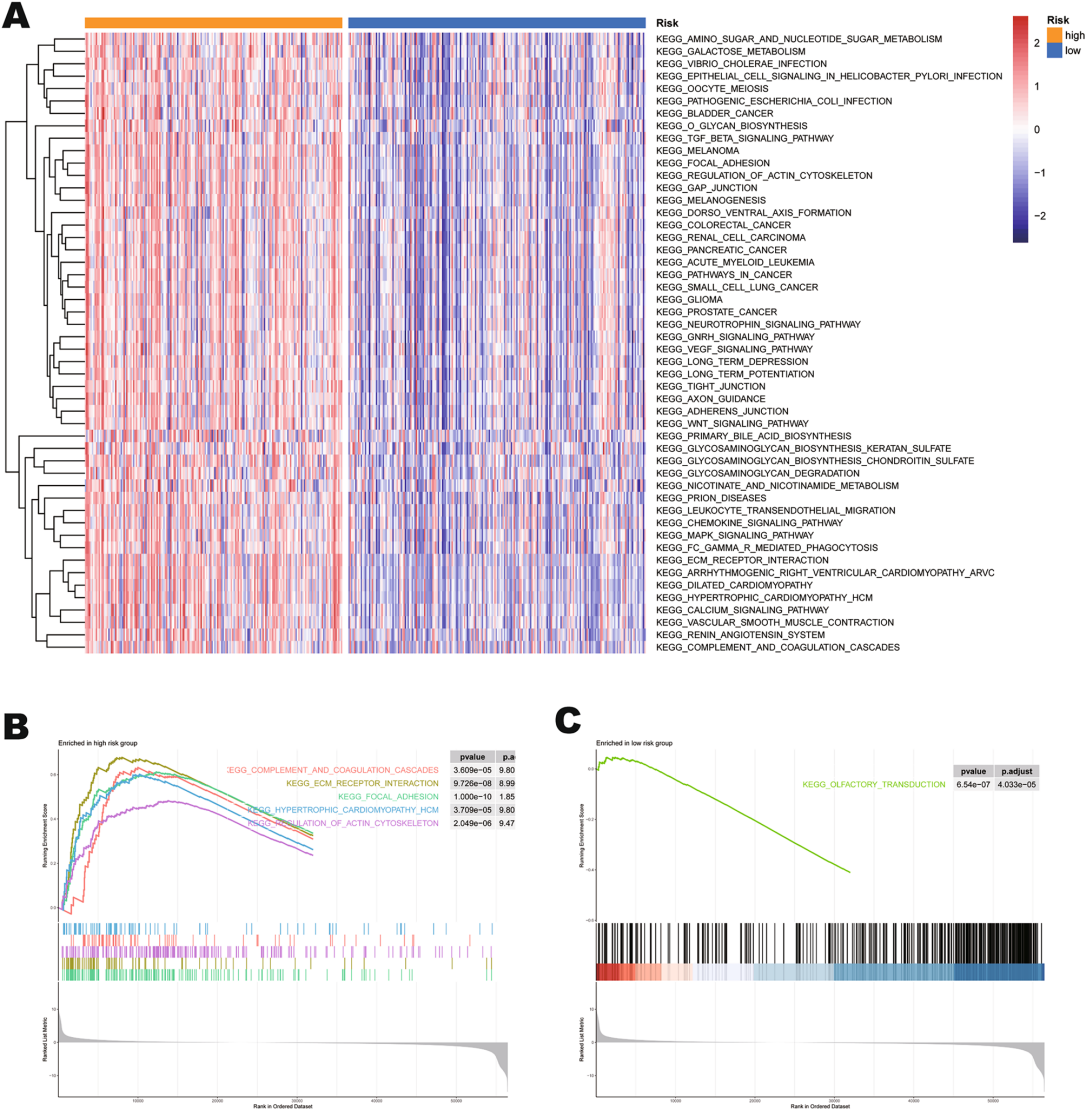
The underlying biological function of mtPCD groups. (**A**) GSVA enrichment analysis based on KEGG pathways. (**B**) GSEA enrichment analysis for the high-risk group based on KEGG pathways. (**C**) GSEA enrichment analysis for the low-risk group based on KEGG pathways.

In order to explore the relevant biological functions and related pathways of the mtPCD groups, we performed enrichment analyses. The results of GSVA functional enrichment analysis showed (Fig. 6A) that the high-risk group was mainly enriched in environmental information processing and cellular process-related pathways (focal adhesion, actin cytoskeleton regulation, tight junction, WNT signaling pathway, TGF-β signaling pathway and PI3K-AKT signaling pathway). In addition, there were multiple tumor (bladder cancer, renal cell cancer, and colorectal cancer) pathways in the high-risk group, which is consistent with our previous analyses and research directions. Subsequently, we performed GSEA analysis on both mtPCD groups to further identify significantly enriched activation pathways. As shown in the figure (Fig. 6B,C), the high-risk group was mainly significant (focal adhesion, ECM-receptor communication and complement coagulation cascades signaling pathways). Whereas in the low-risk group we found only one olfactory transduction related pathway.

### Drug sensitivity evaluation of the mtPCD-related prognostic signature

**Figure 7:**
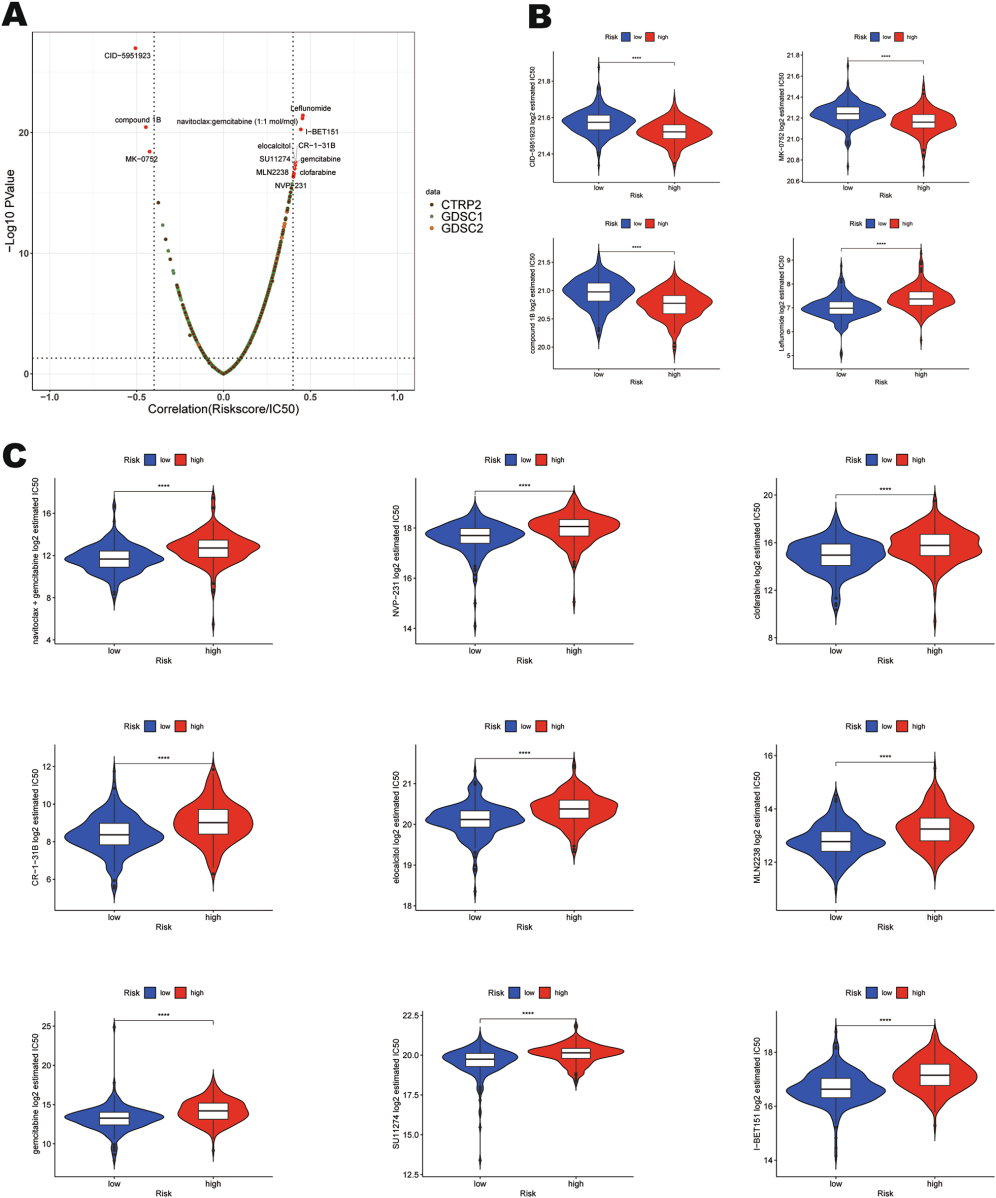
Drug sensitivity evaluation of the mtPCD-related prognostic signature. (**A**) Analyze the relationship between drug IC50 values and mtPCD risk score, and identify 13 candidate drugs. (**B**, **C**) Comparison of drug sensitivity between low-risk and high-risk groups in the TCGA-BLCA cohort.

We examined the relationship between drug IC50 values and mtPCD risk score by spearman analysis (Fig. 7A), and ultimately screened out 13 drugs that showed significant differences. Subsequently, we used the R package "oncoPredic" to calculate the distribution of drug IC50 between high- and low-risk groups. As shown in the figure (Fig. 7B,C), the high-risk group showed higher sensitivity to CID-5951923, compound 1B, and MK-0752. However, compared to the low-risk group, the high-risk group were less sensitive to Leflunomide, navitoclax: gemcitabine (1:1 mol/mol), I-BET151, elocalcitol, CR-1-31B, SU11274, gemcitabine, MLN2238, clofarabine, and NVP-231. In conclusion, these results suggest that high-risk patients can benefit from chemotherapy based on CID-5951923, compound 1B and MK-0752.

### Single-cell analysis for mtPCD-related biomarkers in BLCA

**Figure 8:**
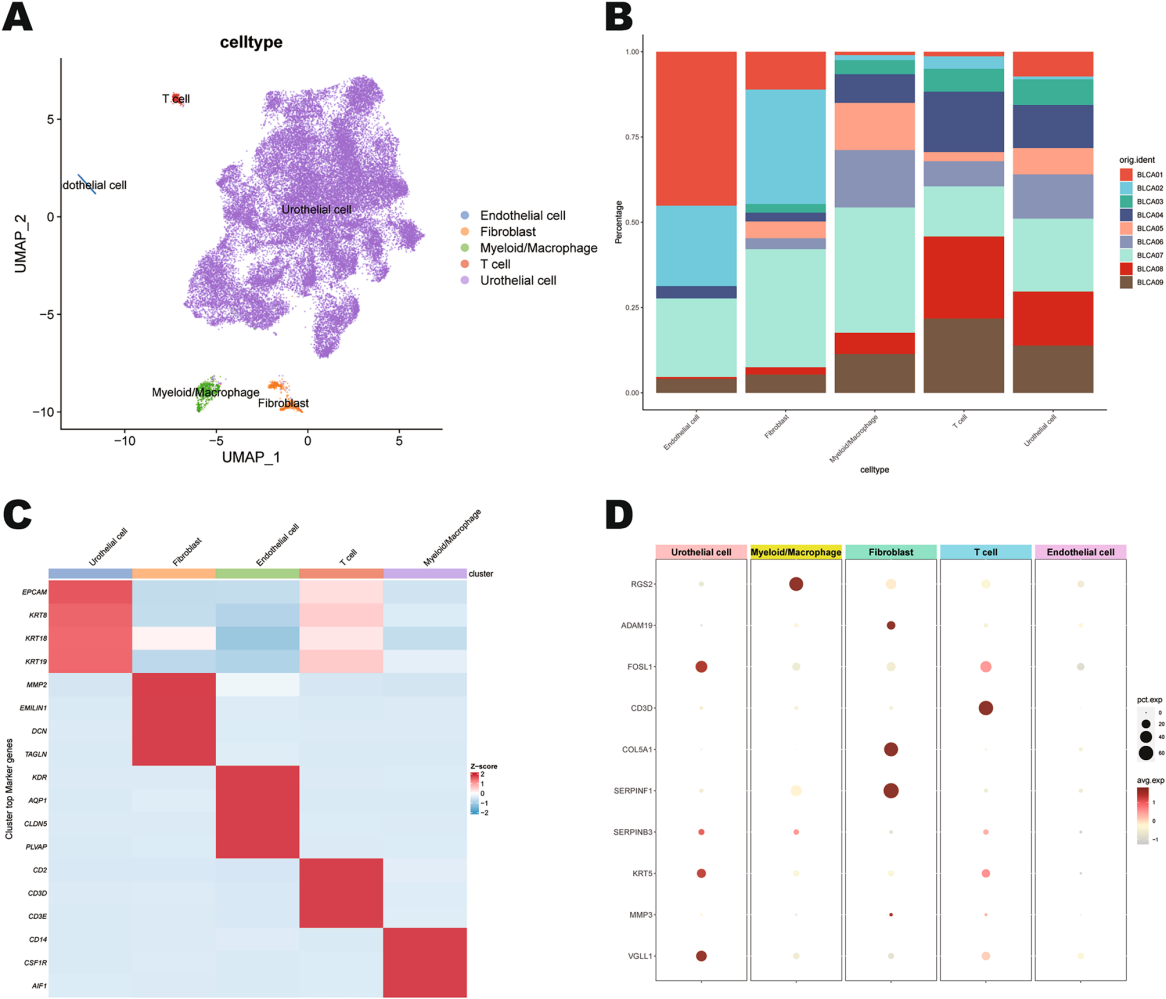
Examining the distribution of signature genes in single-cell sequencing data. (**A**) Annotation of cell populations using marker genes. (**B**) Sample sources and proportions for each cell population. (**C**) Characteristic marker genes of each cell population. (**D**) Dot plot of signature genes expression across different cell populations.

Based on single-cell sequencing datasets (GSE130001 and GSE135337), we clustered the data using the Seurat R package. First, quality control was performed on the datasets (Supplementary Fig. S3A), showing the unique molecular identifier (UMI), gene counts, and mitochondrial genes content for each sample (Supplementary Fig. S3B). The UMI and gene counts exhibited a significant positive correlation (Supplementary Fig. S3C). After integration with the Harmony package, 31 Harmony reductions were used for the subsequent analysis (Supplementary Fig. S3D). Utilizing UMAP for visualization, 5 different cell types were finally identified as endothelial cells, fibroblasts, myeloid/macrophages, T cells and urothelial cells (Fig. 8A). Following that, the origin and proportion of samples for each cell type, along with the marker genes, were presented (Fig. 8B,C). Next, the results of dot plot showed (Fig. 8D) that RGS2 was highly expressed in myeloid/macrophages, fibroblasts, and T cells, and partially expressed in urothelial cells and endothelial cells. ADAM19 and COL5A1 were mainly expressed fibroblasts. FOSL1, KRT5 and VGLL1 were highly expressed in urothelial cells and T cells, and partially expressed in myeloid/macrophages, fibroblasts, and endothelial cells. CD3D is mainly expressed in T cells. SERPINF1 was mainly expressed in fibroblasts and myeloid/macrophages. SERPINB3 was mainly expressed in urothelial cells. MMP3 was mainly expressed in urothelial cells and fibroblasts.

### Immunohistochemistry and RT-qPCR of mtPCD characteristic genes

**Figure 9:**
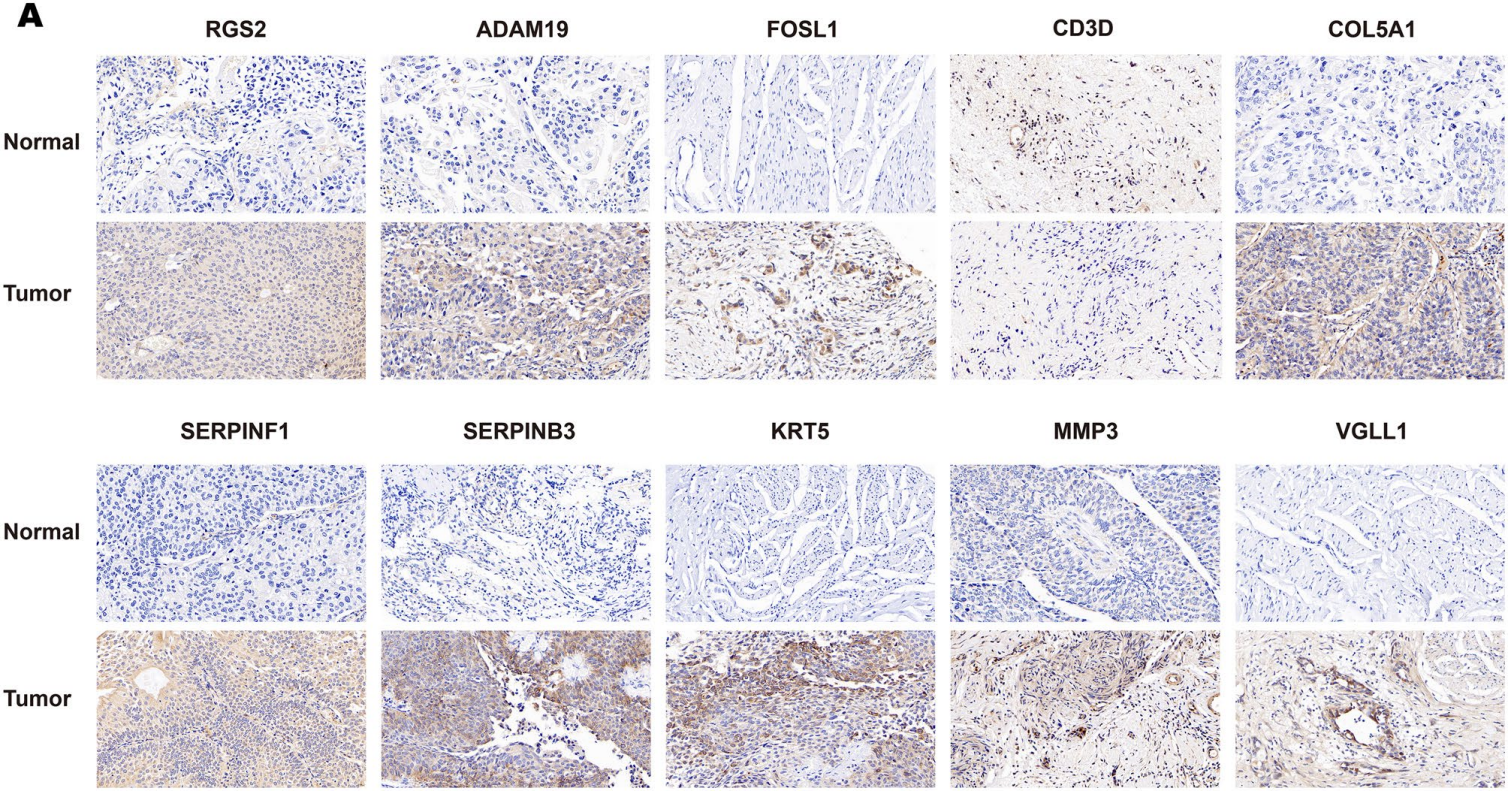
IHC of mtPCD signature genes. (**A**) Differential protein expression of 10 signature genes in MIBC BLCA tumor and normal tissues (scale = 20 μm).

**Figure 10:**
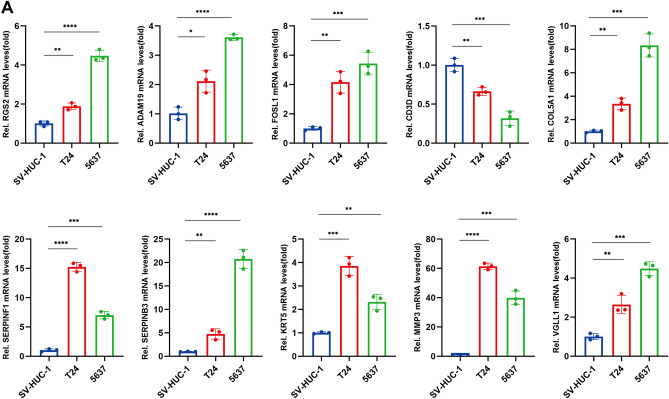
RT-qPCR of mtPCD signature genes. (**A**) Differential mRNA expression of 10 signature genes in tumor tissue and normal tissue.

Previous studies have highlighted the potential of mtPCD as a prognostic indicator for BLCA. To explore the differences in protein expression levels of signature genes between cancerous and adjacent non-cancerous tissues, we performed immunohistochemistry (IHC) staining on paired samples collected from 15 non-muscle-invasive BLCA (NMIBC)and 7 muscle-invasive BLCA (MIBC) cases, respectively, accompanied by clinical information (Supplementary Table 4). Subsequently, representative IHC images were selected based on staining intensity. The MIBC IHC results (Fig. 9A) showed significantly higher protein expression levels of RGS2, ADAM19, FOSL1, COL5A1, SERPINF1, SERPINB3, KRT5, MMP3, and VGLL1 in BLCA samples compared to paired non-cancerous samples. Conversely, CD3D exhibited higher protein expression in adjacent non-cancerous tissues. Consistent results were also obtained in NMIBC (Supplementary Fig. S4A). The predominant expression of CD3D in T cells has been validated through single-cell analysis, providing theoretical support for the IHC findings. Additionally, RT-qPCR validation also confirmed these results, indicating elevated expression of most signature genes in tumor cells. Specifically, apart from CD3D, all identified signature genes exhibited significant upregulation in tumor cells (Fig. 10A).

## Discussion

BLCA is a prevalent urological malignancy, and its incidence is gradually rising globally due to population growth and aging trends^16^. While ICI have brought new breakthroughs in the treatment of BLCA, only about 25% of patients benefit from this therapy. It is noteworthy that even among responders, long-term therapeutic intervention is still required^17^. Consequently, there is a pressing need for ongoing refinement of treatment strategies, accurate prediction of immunotherapeutic responses, and the exploration of innovative therapeutic modalities to address the complex management challenges posed by BLCA. Cell death serves a dual role in cellular homeostasis, ensuring the turnover of damaged cells while also exerting control over the proliferation and survival of tumor cells^18^. Tumor cells continuously evade the PCD pathway by evolving various mechanisms, highlighting the significance of PCD in tumor progression. In addition, the PCD pathway is also closely related to the tumor immune surveillance function^19^. From this perspective, enhancing the regulation of PCD in tumors may hold promise for anti-cancer therapy.

In this study, we initially identified 25 candidate genes associated with prognosis through ssGSEA, differential expression analysis and univariate analysis. Subsequently, we successfully identified 10 core mtPCD genes (RGS2, ADAM19, FOSL1, CD3D, COL5A1, SERPINF1, SERPINB3, KRT5, MMP3, and VGLL1) using the LASSO regression analysis method, from which an mtPCD signature was established. Survival analysis revealed that the mtPCD score served as an adverse indicator of OS, with ROC curves demonstrating the high accuracy and stability of the mtPCD signature across TCGA-BLCA, GSE13507, and GSE32849 cohorts. These findings collectively highlight the promising clinical utility of mtPCD. In addition, we searched for many published models containing various combinations of genes with various biological functions. Then, we screened the comparable models for final comparison. Encouragingly, our models exhibited significant advantages in each cohort, underscoring the superior predictive performance of our model in determining BLCA prognosis.

TIME, an essential factor influencing tumorigenesis and progression, holds the potential to inspire novel therapeutic approaches for tumors through its analysis^20^. TIME is often accompanied by the development of immune infiltrates, which are immune cells that can both support and impede tumor therapeutic efficacy, and which vary in their activation status and localization in TIME^21^. Our study further explored the relationship between mtPCD and TIME in BLCA. It was observed that the low-risk group had higher levels of CD8 + T cells, CD4-activated memory T cells, follicular helper T cells and regulatory T cells infiltration. In contrast, high levels of M0 and M2 macrophages infiltration were observed in the high-risk group. In the high-risk group, three immune checkpoint genes CD274 (PD-L1), PDCD1LG2 (PD-L2) and HAVCR2 (TIM-3), along with five immunosuppressive chemokines (IL10, PTGER2, PTGER3, TGFB1, TGFB2, and TGFB3), exhibit significantly elevated expression levels. M0 macrophages represent a quiescent state capable of differentiating into M1 and M2 macrophages. Of particular note, these five immunosuppressive molecules are secreted by M2 macrophages and inhibit immune function through various biological processes, thereby promoting tumor proliferation^22^. Meanwhile, the three immune checkpoint genes play crucial roles in T cell activation and significantly influence responses to immunotherapy^23^. In addition, tumor immune exclusion scores were lower in the low-risk group compared to the high-risk group, and the results of immune infiltration also indicated higher levels of CD8 + T cells in the low-risk group. The results of enrichment analysis indicate that the high-risk group is enriched in the PI3K-AKT signaling pathway and TGF-β signaling pathway. The study demonstrates a bidirectional regulatory relationship between the PI3K-AKT-mTOR pathway and PD-L1, which is observed in both tumor cells and TIME^24^. TGF-β impacts CD8 T cells and consequently influences the entire TIME by inhibiting the expansion and differentiation of stem cell-like CD8 T cells^25^. These results all suggest that the high-risk group may achieve immune escape of tumor cells by preventing T-cell infiltration, leading to poorer immunotherapy response and prognosis. Therefore, significant differences in immune status exist between different groups, where high-risk group demonstrate tumor-associated macrophage-induced immune suppression compared to low-risk group. Moreover, immune therapy response scores among risk groups indicate that patients in the low-risk group were more likely to benefit from inhibition of immune checkpoints (CTLA-4 and PD-1), thus reinforcing our findings. Drug sensitivity analyses identified three agents, namely CID-5951923, compound 1B, and MK-0752, exhibiting heightened efficacy in the high-risk group. These drugs provide new options for potential treatment in the high-risk group, while further emphasizing the need to develop personalized therapeutic strategies for BLCA patients based on the molecular signature of mtPCD.

RGS2 belongs to the family of regulators of G-protein signaling proteins, consisting of 120 amino acids, primarily mediating the activity of GTPase-activating proteins^26^. Cho’s study revealed that RGS2 enhances dormancy cancer cells’ anti-apoptotic ability induced by endoplasmic reticulum stress through modulation of ATF4 expression, correlating with tumor recurrence and chemotherapy resistance^27^. FOSL1, a transcription factor of the Fos gene family, is closely associated with inflammation and apoptosis and widely employed in various disease studies^28^. Recent studies indicate that in neuronal injury models, FOSL1 inhibits autophagy and promotes inflammation and cell apoptosis via the AMPK signaling pathway, highlighting its significant role in the regulation of apoptosis^29^. SERPINF1 inhibits endothelial cell migration and promotes apoptosis through the P53 pathway and exogenous signaling of CD95L and TRAIL, thereby suppressing tumor angiogenesis^30,31^. Interestingly, SERPINF1 exhibits bidirectional regulatory effects in tumor development. According to Li’s research^32^, intracellular SERPINF1 induces free fatty acid accumulation, promoting hepatocellular carcinoma cell growth, whereas secreted SERPINF1 traditionally exerts anticancer effects. SERPINB3 is overexpressed in various tumors due to its anti-apoptotic properties, garnering significant attention^33^. Elevated levels of SERPINB3 in P66shc-downregulated tumors inhibit Caspase-8 activity, rendering tumor cells more susceptible to necroptosis^34^. Located on chromosome 12, KRT5 is associated with several skin diseases and has recently been implicated in tumorigenesis. Particularly in BLCA, combined detection of KRT5 and KRT20 is considered a potential alternative to IHC for predicting prognosis in MIBC^35^. KRT5 has also been proposed as a novel candidate biomarker and therapeutic target in prostate cancer^36^. Frieling^37^ discovered that MMP3 promotes bone metastasis of prostate cancer both in vitro and in vivo and is considered a therapeutic target to inhibit the occurrence and metastasis of breast cancer^38^. VGLL1 has been proven to play a crucial role in various cancer types, with its high expression often associated with lower overall survival. The phosphorylation of VGLL1 in the TGF-β/ERK/RSK2 signaling pathway plays a key role in MMP9-mediated malignant gastric cancer, emphasizing the potential of VGLL1 as a targeted therapy for gastric cancer^39^. COL5A1 is highly expressed in several tumors with high malignancy, particularly in triple-negative breast cancer. It promotes M2 macrophage polarization to drive the TGFβ/Smad3/COL5A1 signaling pathway, leading to tumor drug resistance^40^. ADAM19 is a cell surface glycoprotein that is overexpressed in lung and kidney inflammation as well as tumors. Therefore, targeted inhibition of its expression may be crucial for therapy^41^. CD3D is a characteristic molecule on the surface of T cells, primarily involved in the activation and regulation of the immune system. It is closely associated with immune checkpoints and immune cell infiltration. Hence, high expression of CD3D typically indicates better clinical prognosis for cancer patients^42^. Previous studies have emphasized the close association of these signature genes with tumorigenesis, primarily through PCD affecting the progression of tumors. These theories further substantiate our research findings.

In summary, the mtPCD score in our study provides a feasible supplementary option for current BLCA prognosis assessment criteria, with its superior performance being comprehensively validated. In BLCA patients with mtPCD features, ICI therapy demonstrates significant potential. Therefore, screening for mtPCD features contributes to identifying the most beneficial patient population. Meanwhile, drug sensitivity analysis reveals three potential chemotherapeutic agents, offering new avenues for personalized BLCA treatment. Thus, mtPCD score will play a pivotal role in guiding both chemotherapy and immunotherapy for BLCA in the future. Nonetheless, the mtPCD score needs further validation if it is to be used as an established standard in clinical practice. In particular, as a prognostic biomarker, simplifying its identification and separation, unifying assessment criteria, and reducing financial and manpower consumption will be significant challenges. Additionally, the mechanisms by which the mtPCD score impacts tumor immune infiltration remain incompletely understood, necessitating extensive basic experiments that could potentially offer new opportunities for immunotherapy in BLCA. However, this research faces several methodological limitations. One of the primary constraints is its heavy reliance on retrospective datasets from public databases. While preliminary validation has been conducted, comprehensive validation across more cohorts cannot be guaranteed.

## Methods

### Data source

Bulk RNA-seq data containing 403 BLCA samples with clinical survival information was obtained from The Cancer Genome Atlas (TCGA) database^43^. Microarray expression data and corresponding clinical data were obtained from GSE13507^44^ containing 165 BLCA patients from the GPL6102 platform and GSE32894^45^ containing 224 BLCA patients from the platform GPL6947 in the GEO database (http://www.ncbi.nlm.nih.gov/geo). Furthermore, the scRNA-seq dataset GSE130001^46^ of 2 BLCA samples and GSE135337^47^ of 7 BLCA samples were obtained from the GEO database. The IMvigor 210 cohort, which included 168 patients with urothelial carcinoma treated with anti-PD-L1 therapy, was obtained from “IMvigor210CoreBiologies”^48^ package. Through literature search, we obtained the mtPCD genes set from the Supplementary file of the relevant literature, which includes 18 PCD patterns and key regulatory genes as well as mitochondria-related genes^49^.

### Evaluation of differentially expressed mtPCD related genes in BLCA

ssGSEA (single-sample gene set enrichment analysis) was performed to estimate the relative enrichment score of mtPCD genes set in the TCGA-BLCA samples, using the GSVA package. “Surv_cutpoint” function, was used to calculate the optimal cutpoint value to distinguish between the high and low mtPCD score groups. Kaplan–Meier (K-M) survival curves and Logrank tests, bases on the “survival” package served to analyze and compare the survival rates of low and high mtPCD score to determine whether mtPCD genes are related to poor prognosis in bladder cancer. Differentially expressed mtPCD related genes (mtPCDRGs) between the high and low mtPCD score groups in the TCGA-BLCA database were acquired using the “limma’’^50^ package (|Log2FC|> 1 and adj.*P* < 0.05). Volcano maps of these mtPCDRGs were plotted.

### Construction and validation the prognostic signature

To obtain candidate genes related to prognosis, univariate Cox analysis was performed on the above mtPCDRGs. After using the “sva”^51^ package to remove batch effects from various databases, co-expressed candidate genes are used for the next analysis. The 403 samples in the TCGA-BLCA database were divided into training and testing sets in the ratio of 1:1, with 203 cases as the training set and 200 cases as the testing set. The GSE13507 and GSE32894 database served as validation set and additional validation set. Candidate mtPCDRGs with *p*-value < 0.05 were included in the least absolute shrinkage and selection operator (LASSO) regression analysis, which was performed with the “glmnet”^52^ package. A mtPCD risk score was calculated for each BLCA sample in the training set under the following formula:

$$\text{riskScore}={\sum }_{i=1}^{n}[exp\left(\text{gene}\right)*coef(\text{gene})]$$

This was subsequently categorized into high and low mtPCD risk group based on the median training mtPCD riskScore and generalized to the testing and validation groups. K-M survival curves and receiver operating characteristic (ROC) curves were plotted to inspect the prognostic performance. The Wilcoxon test were used to analyzed differences in mtPCD riskScore between the different clinical indicator subgroups. Univariate and multivariate Cox regression analyses were performed to determine whether the mtPCD riskScore independently served as a significant prognostic indicator. Furthermore, a dynamic nomogram was established to predict the survival rates of patients with BLCA, based on “DynNom” package. Calibration curves, ROC analysis and concordance index (C-index) were used to evaluate the predictive performance of nomogram.

### Comparison of published signatures in BLCA

By searching for PubMed (https://pubmed.ncbi.nlm.nih.gov/), we collected 35 published mRNA signatures of BLCA for performance comparison with mtPCD riskScore. Due to limitations in the validation group, the lncRNA signatures were discarded. These collected signatures were based on various algorithms, including LASSO, RSF, and Cox proportional-hazards model. We collected the genes and their corresponding coefficients composed of these signatures. If specific coefficients were not provided, we reconstructed them with Cox model. Finally, a forest plot was used to display the 95% confidence interval of the C-index for each signature, as well as the statistical significance of the difference in mtPCD riskScore compared to other signatures (two-sided Student T test).

### Enrichment analysis for mtPCD signature

To reveal their underlying biological functions and potential mechanisms associated with mtPCD riskScore groups, we performed Gene Ontology (GO), Kyoto Gene Encyclopedia and Genomic Pathway Enrichment Analysis (KEGG) and gene set enrichment analysis (GSEA) using the “clusterProfiler”^53^ package. We also used the “GSVA”^54^ packages to perform GSVA algorithm to examine the biological activities of the different mtPCD riskScore groups. The gene sets of “c2.cp.kegg.v2023.1.Hs.symbols.gmt” and “c5.go.v2023.1.Hs.symbols.gmt” were downloaded from the MSigDB (https://www.gsea-msigdb.org/gsea/msigdb/index.jsp) database for running GSEA and GSVA analysis.

### TIME and Immunotherapy response prediction

To evaluate proportion of immune cell infiltration in BLCA patients from the TCGA database, we utilized CIBERSORT algorithms, based on the “IOBR” package. The correlation between mtPCD riskScore and immune cell infiltration was analyzed by the Spearman correlation coefficient. The IPS of TCGA-BLCA was downloaded from The Cancer Immunome Atlas (TCIA) database (https://tcia.at/home)^55^. T cell exclusion score was obtained from TIDE (http://tide.dfci.harvard.edu/) website^56^. To assess the effectiveness of our signature in predicting response to anti-PD-L1 agent (atezolizumab), mtPCD riskScore of samples in IMvigor 210 was computed based abovementioned formula. Wilcoxon test was used to analyzed differences in mtPCD riskScore between the CR/PR and SD/PD subgroups.

### Chemotherapy drug sensitivity analysis

Based on expression and drugs sensitivity data from the GDSC1,GDSC2 and CTRP2 database, the “oncoPredict”^57^ package was used to investigate variations in the IC50 values of drugs among BLCA patients. In order to screen potential drugs, Spearman's analysis was used to examine the relationship between drug IC50 values and mtPCD riskScore. We examined the variations in IC50 between the high and low riskScore groups for those whose correlation values were higher than 0.4.

### Single-cell analysis

In this study, the scRNA-seq data of the GSE130001 and GSE135337 dataset was processed with the “seurat”^58^ package. First, cells with less than 200 UMI, genes detected in less than 3 cells, and cells with expressed genes fewer than 500 or more than 6000 were excluded, the proportion of mitochondria genes was limited to less than 10%. Additionally, cells with a UMI to gene ratio greater than 0.8 were selected. Next, we normalized all samples, removed remove confounding sources of UMI, mitochondrial percentage and call cycle by SCTransform^59^. Harmony^60^ algorithm was used to remove the batch effect. Principal component analysis (PCA) was then performed to identify significant harmony reduction. After the initial 31 harmony reductions were selected, cells were clustered using uniform manifold approximation and projection (UMAP) (resolution = 0.5). The cell types were annotated by the following markers: Urothelial cell (EPCAM, KRT8, KRT18, KRT19), Fibroblast (MMP2, EMILIN1, DCN, TAGLN), Endothelial cell (KDR, AQP1, CLDN5, PLVAP), T cell (CD2, CD3D, CD3E), Myeloid/Macrophage (CD14, CSF1R, AIF1). Subsequently, the expression levels of the mtPCD riskScore biomarkers in different cell groups were analyzed and visualized.

### Cell lines and cell culture

All cell lines were obtained from the Type Culture Collection of the Chinese Academy of Sciences (Shanghai, China). Human immortalized uroepithelial (SV-HUC-1) cell line was cultured with Ham’s F-12 K (HyClone, China)/10% fetal bovine serum (Gibco, Australia) media, while BLCA cell lines (5637, T24) were cultured with RPMI 1640 (HyClone, China)/10% fetal bovine serum media. All cells were cultured in an incubator with 5% CO2 at 37 °C.

### Quantitative real-time PCR

Total RNA was extracted from tissues and cell lines using the TRIzol reagent (Thermo Fisher Scientific, USA, USA). cDNA was synthesized from the total RNA using PrimeScriptTM RT Reagent Kit (TaKaRa, Japan). RT-qPCR was performed using TB Green PCT Master Mix (Akara, Japan) on a LightCycler 96 instrument. GAPDH was used for experimental reference. Each sample was measured at least three times, and the mRNA levels were quantified using the ΔΔCt method. All PCR primers were purchased from Sangon Biotech (Shanghai, China), and sequences are listed in Supplementary Table S5.

### Immunohistochemistry

The clinical samples were paraffin-embedded. IHC staining was performed on 5 μm-thick sections. Sections were deparaffinized, rehydrated, and underwent antigen retrieval. After blocking with 5% goat serum at ambient temperature for 1 h, the slides were incubated with primary antibodies at 4 °C overnight, followed by secondary antibody incubation. Sections were reacted with diaminobenzidine (DAB) for 2 min, washed with tap water, and then stained with hematoxylin. Finally, the sections were observed and photographed under a microscope.

Primary antibodies used in IHC are listed as follows: RGS2(CUSABIO CSB-PA826561, 1:100); ADAM19(BOSTER BA3839, 1:400); FOSL1(BOSTER M03927-1, 1:50); CD3D (Affinity Biosciences DF6370, 1:200); COL5A1(CUSABIO CSB-PA005748KA01HU, 1:100); SERPINF1(BOSTER BA1348-1, 1:400); SERPINB3 (Affinity Biosciences DF7335, 1:100) KRT5(CUSABIO CSB-PA173763, 1:100); MMP3(CUSABIO CSB-PA07449A0Rb, 1:100) VGLL1(Proteintech 10124-2-AP, 1:100).

### Statistical analyses

For statistical analysis and graphical visualization of the data, we used R (version 4.2.1) and GraphPad Prism 9.0 software. A significance level of *p* < 0.05 was considered statistically significant. *P < 0.05, **P < 0.01, ***P < 0.001, ****P < 0.0001. ns, no significance.

### Ethical approval

The collection of tissue specimens and experimental methods were approved by the Ethics Committee of Renmin Hospital of Wuhan University. Informed consent was obtained from all patients. All methods were performed in accordance with the relevant guidelines and regulations.

### Supplementary Information

Supplementary Tables.

Supplementary Figures.

## Data Availability

Data availability Public data used in this work can be acquired from the TCGA (https://portal.gdc.cancer.gov/) and GEO (https://www.ncbi.nlm.nih.gov/geo/). More detailed data is available from the corresponding author on reasonable request.
