# Supplementary material for: A novel signature constructed by mitochondrial function and cell death-related gene for the prediction of prognosis in bladder cancer
Source: Sci Rep. 2024 Jun 25;14:14667. doi: 10.1038/s41598-024-65594-0 (PMC11199696; doi:10.1038/s41598-024-65594-0)
Supplement: Supplementary file 2 — Supplementary Figures. [file 41598_2024_65594_MOESM2_ESM.docx]

**SUPPORTING INFORMATION**

**A novel signature constructed by mitochondrial function and cell death-related gene for the prediction of prognosis in bladder cancer**

Zhiwei Yan^1,2†^, Yunxun Liu^1,2†^, Minghui Wang^1,2†^, Lei Wang^1,2*^, Zhiyuan Chen^1,2*^, and Xiuheng Liu^1,2*^


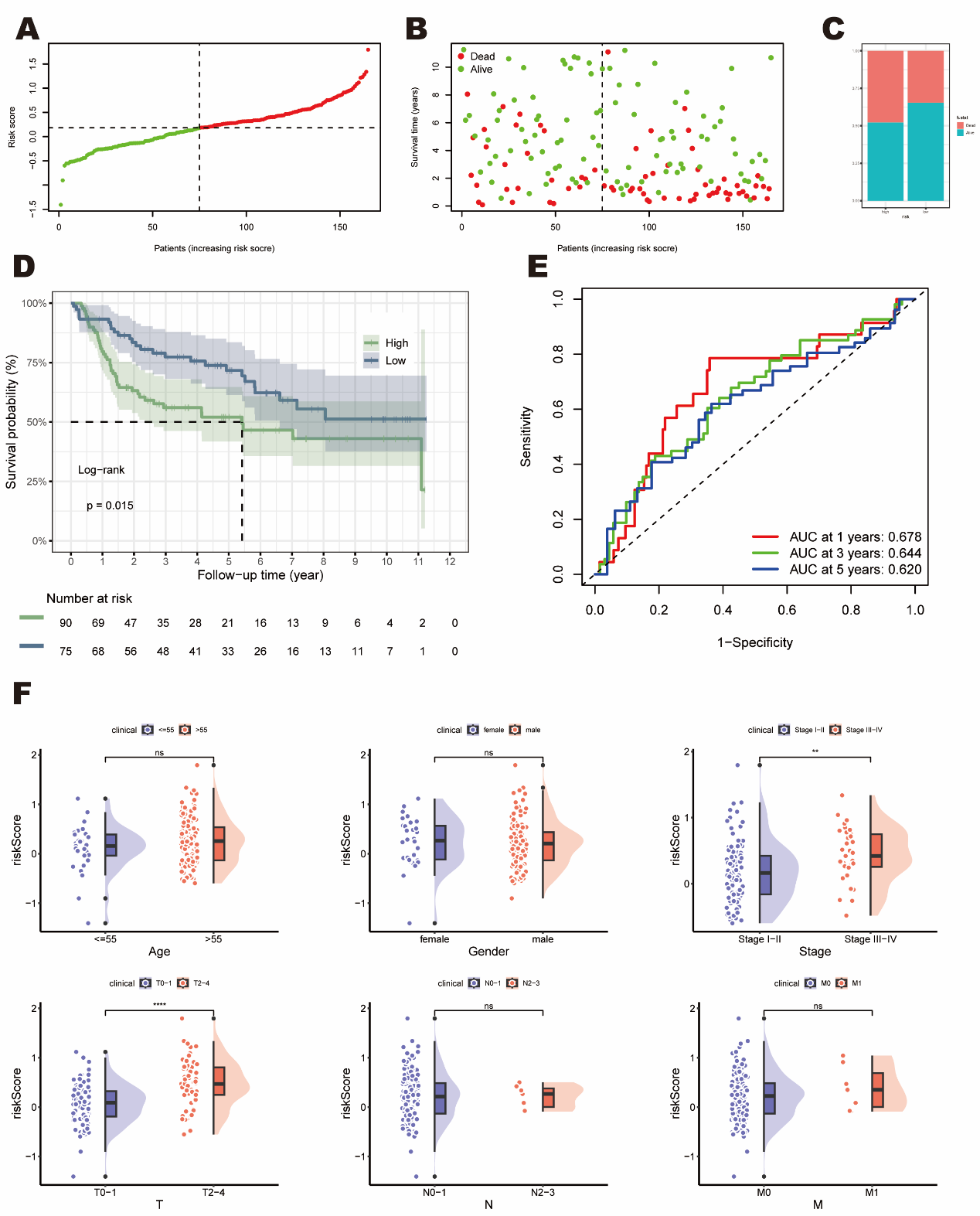


**Fig. S1** Validation of the prognostic signature and analysis of clinical relevance based on the GSE13507 cohort. A-C Distribution of patients and the proportion of individuals in different survival states. D Kaplan Meier curves. E ROC curves at 1-, 3-, and 5- year in the GSE13507 cohort, with respective AUC of 0.678, 0.644, and 0.620. F Demonstrates the risk score distribution based on different clinical pathological features


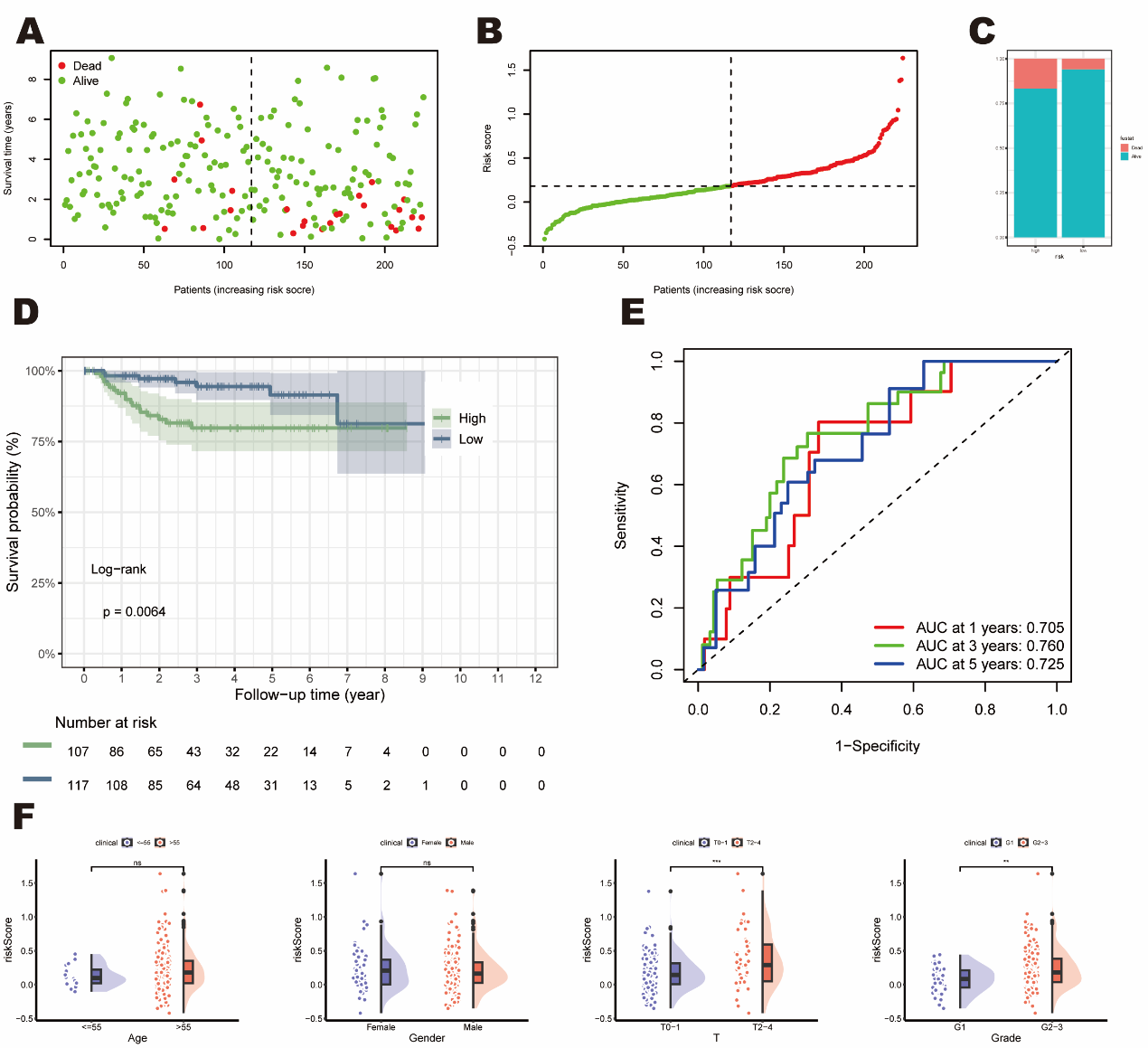


**Fig. S2** Validation of the prognostic signature and analysis of clinical relevance based on the GSE32894 cohort. A-C Distribution of patients and the proportion of individuals in different survival states. D Kaplan Meier curves. E ROC curves at 1-, 3-, and 5- year in the GSE32894 cohort, with respective AUC of 0.705, 0.760, and 0.725. F Demonstrates the risk score distribution based on different clinical pathological features


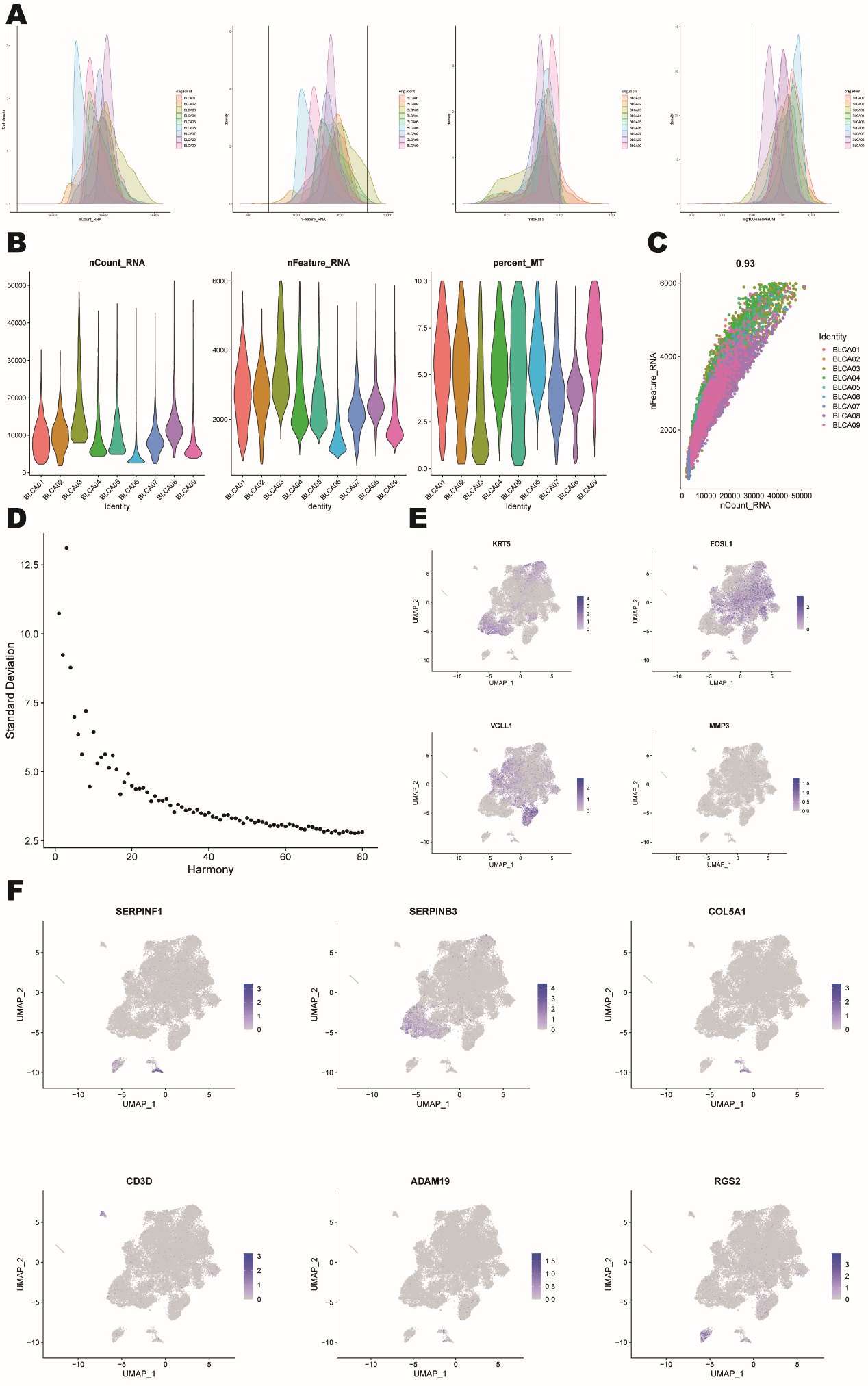


**Fig. S3** Single-cell analysis. A Quality control for datasets GSE130001 and GSE135337. B UMI, number of genes, and mitochondrial genes content for each sample. C Scatter plot illustrating a positive correlation between UMI and number of genes. R=0.93. D Gravel plots derived from PCA results. E-F Expression of signature genes across different cell populations


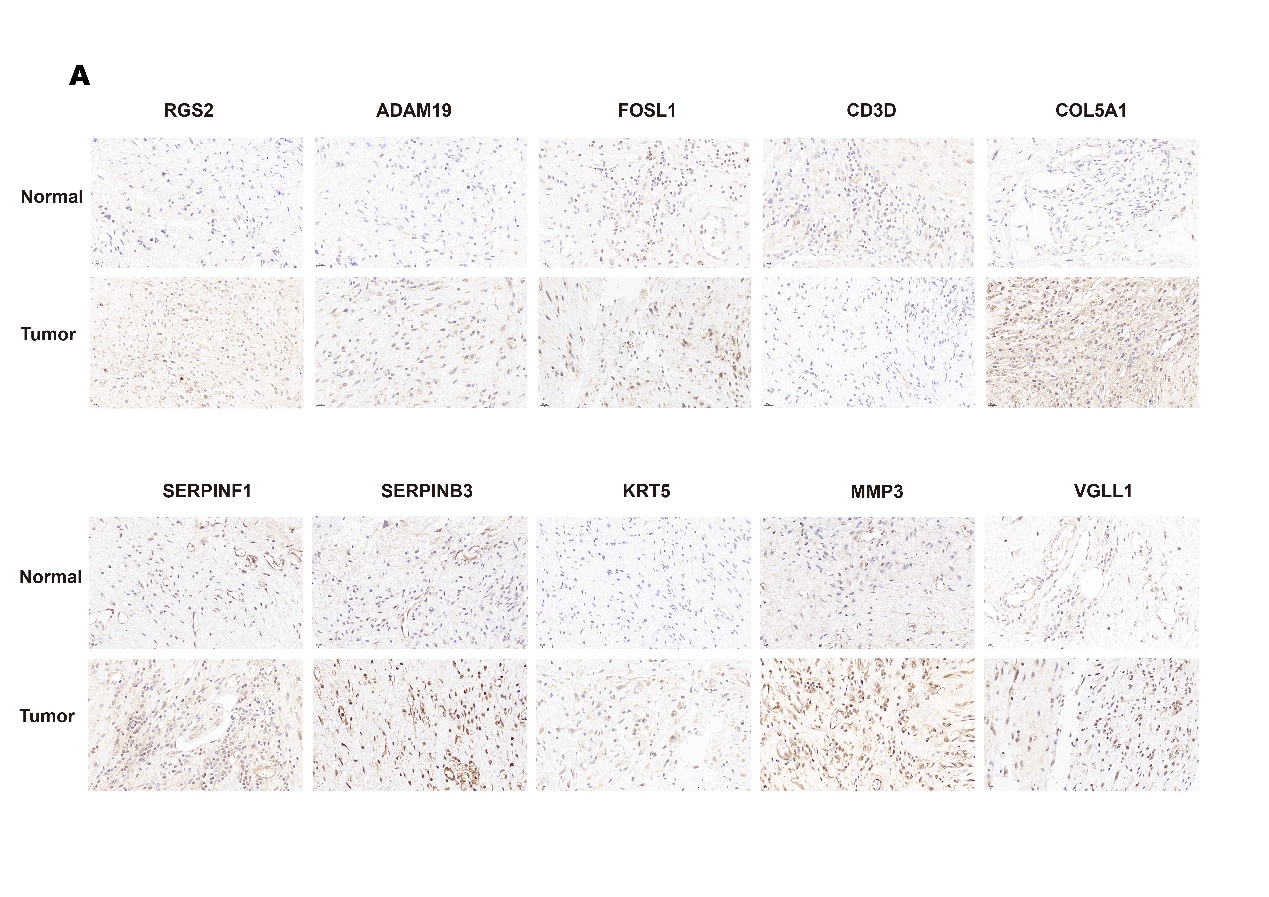


**Fig. S4** IHC of mtPCD signature genes. A Differential protein expression of 10 signature genes in NMIBC tumor and normal tissues (scale = 20μm)
